# Real-Time Small Retail Product Detection in Low-Light Intelligent Cabinets Under Complex Backgrounds

**DOI:** 10.3390/s26103264

**Published:** 2026-05-21

**Authors:** Moushiqi Yang, Junjie Cai, Yuanyuan Yang, Jian Chen, Kai Xie

**Affiliations:** 1 Future Technology Academy, Yangtze University, Jingzhou 434023, China; 2023008598@yangtzeu.edu.cn; 2 School of Physics and Optoelectronic Engineering, Yangtze University, Jingzhou 434023, China; 2024006861@yangtzeu.edu.cn; 3 School of Mathematics and Computer Science, Wuhan Polytechnic University, Wuhan 430023, China; 13018051090@163.com; 4 College of Computer and Information Science, Southwest University, Chongqing 400715, China; cj15727181945@email.swu.edu.cn; 5 School of Electronic Information and Electrical Engineering, Yangtze University, Jingzhou 434023, China

**Keywords:** retail product detection, low-light detection, samll object detection, multi-scale feature fusion, real-time detection, embedding deployment

## Abstract

Intelligent retail cabinets require accurate and real-time detection of small retail products in complex environments, particularly under low-light conditions and large-scale variations. However, existing object detection methods often suffer from insufficient feature representation and unstable performance in small-object retail commodity recognition tasks under low illumination and complex backgrounds. To address these challenges, this paper proposes a real-time small retail product detection framework based on YOLOv26 for low-light intelligent cabinet environments, aiming to improve detection accuracy, robustness, and deployment efficiency. A C3k2-enhanced multi-scale feature extraction module is introduced to strengthen feature representation for small retail products, while a novel detection head integrates minimum-resolution feature layers and an Efficient Multi-scale Attention (EMA) mechanism to enhance feature fitting ability under low-light conditions. In addition, convolution-based downsampling and Content-Aware ReAssembly of Features module (CARAFE) is adopted to improve feature fusion efficiency and reduce computational overhead. Experimental results on the RPC commodity dataset and the 6th Commodity Recognition Competition dataset demonstrate that the proposed method achieves improved detection performance compared with baseline models, with a 0.9% increase in Recall and a 0.2% improvement in mean Average Precision at IoU threshold 0.50 (mAP@50) while maintaining competitive mean Average Precision averaged over IoU thresholds from 0.50 to 0.95 (mAP@50-95). While the GFLOPS value rose from 5.8 to 6.3, deployment on the Jetson Nano platform achieves 25 FPS, demonstrating real-time detection capability in intelligent retail environments. The proposed framework provides a reliable and deployable solution for small retail product detection in low-light intelligent cabinet systems.

## 1. Introduction

With the rapid development of mobile payment and computer vision technologies, vision-based Unmanned Vending Machines (UVMs) have gradually become an important component of intelligent retail systems. Compared with traditional vending solutions, vision-driven UVMs provide higher flexibility, lower hardware costs, and improved user experience, making them suitable for deployment in various retail environments. However, existing retail solutions, including RFID-based systems [1], gravity-sensing systems, and mechanical vending devices, often suffer from high manufacturing costs, complex maintenance, and limited scalability. Therefore, developing a low-cost, high-precision, and real-time visual object detection framework suitable for embedded deployment has become a key research focus in intelligent retail.

In practical retail cabinet environments, products typically exhibit multi-scale distributions, visual similarity, and significant illumination variations, especially under low-light intelligent cabinet conditions. These challenges make retail commodity recognition a complex multi-class object detection problem. Object detection methods are generally categorized into two-stage and one-stage frameworks. Two-stage detectors, such as R-CNN-based approaches [2], Fast R-CNN, and Faster R-CNN [3], achieve high detection accuracy by generating region proposals followed by classification and regression. However, their computational complexity limits real-time deployment on embedded devices. In contrast, one-stage detectors directly perform localization and classification in a unified framework, significantly improving inference speed while maintaining competitive accuracy, making them more suitable for real-time intelligent retail applications.

Recent object detection models include SSD [4], RetinaNet [5], FCOS [6], DETR [7], RT-DETR [8], Deformable DETR [9], CenterNet [10], and YOLO-based frameworks. Among them, YOLOv26 [11] is a recent one-stage detector that achieves a favorable balance between detection accuracy and computational efficiency through end-to-end training and efficient feature fusion mechanisms, making it suitable for embedded retail applications. Common backbone networks in object detection include VGG [12], ResNet [13], MobileNet, and ShuffleNet, while feature fusion architectures typically adopt Feature Pyramid Networks (FPN) and Spatial Pyramid Pooling modules such as SPP/SPPF [14] to enhance multi-scale representation.

Despite these advances, detection performance in retail cabinet environments still degrades due to small object sizes, illumination variations, and complex visual backgrounds, especially under low-light conditions. To address these issues, previous studies have proposed various improvement strategies. Wang et al. [15] introduced generative adversarial networks (GANs) to enhance small-object feature representation; Dong et al. [16] applied super-resolution reconstruction and gating mechanisms to improve discriminative information for small targets; Deng et al. [17] integrated CBAM, ASFF, and EIoU into YOLOv11 to improve detection performance in automated checkout systems. However, most existing methods are designed for general-purpose datasets and lack targeted optimization for low-light intelligent retail cabinet environments, resulting in limited robustness and high computational overhead in embedded deployment scenarios.

Motivated by these challenges, this paper proposes a real-time small retail product detection framework based on YOLOv26 for low-light intelligent cabinet environments, aiming to improve detection accuracy, robustness, and deployment efficiency under complex retail conditions. The main contributions of this study are summarized as follows:

1. **Real-Time Small Retail Product Detection Framework:** A real-time small retail product detection framework based on YOLOv26 is developed, in which a C3k2-enhanced module is introduced into the backbone to strengthen multi-scale feature representation and improve robustness to small retail products under low-light intelligent cabinet environments.
2. **EMA-Based Feature Fusion and Detection Head Design:** An Efficient Multi-scale Attention (EMA) mechanism is integrated into the detection head, and a Conv-based downsampling layer and a CARAFE upsamping module is introduced to enhance feature fusion efficiency and improve detection performance under low-light and complex retail environments.
3. **Real-Time Embedded Deployment:** The proposed model is optimized for edge-device environments and deployed on the Jetson Nano platform, achieving real-time inference at 25 FPS while maintaining stable detection accuracy under limited computational resources.

## 2. Related Work

### 2.1. Small Object Detection

Small-object detection in scenarios such as UAV aerial imagery and infrared sensing remains a challenging task due to the extremely small pixel footprint of targets and the low signal-to-noise ratio. Recent studies have largely focused on improving YOLO-based detection frameworks to enhance multi-scale feature representation and spatial attention mechanisms. Wang et al. proposed CF-YOLO, which integrates a contextual feature fusion module into the YOLOv11 backbone to improve small-object detection performance in UAV imagery [18]. For infrared small-target detection, Tang et al. developed the Irstd-YOLO framework, introducing an improved feature pyramid structure and attention mechanism to suppress background clutter [19]. Yue et al. combined super-resolution preprocessing with YOLO and proposed YOLO-MST, demonstrating that upsampling low-resolution infrared inputs can significantly improve detection accuracy [20]. Similarly, Zhang et al. proposed PARE-YOLO, which incorporates cross-layer multi-scale attention to better capture fine-grained details in aerial images [21]. These YOLO-based improvements highlight the importance of customized feature enhancement and multi-scale feature fusion for accurate localization of small targets.

### 2.2. Low-Light Image Enhancement

Image degradation under low-light conditions significantly affects the performance of conventional object detection models. To address this issue, Pan et al. proposed NID-DETR, a detection transformer designed specifically for dark environments, which integrates illumination-invariant feature representations to improve robustness [22]. In addition, Wang et al. introduced a low-light image enhancement method based on the physical dehazing model, which restores image contrast and color fidelity without requiring paired training data [23]. These approaches demonstrate a growing trend of combining physical priors with deep learning techniques to mitigate the adverse impact of low-light conditions on downstream detection tasks.

### 2.3. Edge Device Deployment

Deploying real-time object detection on resource-constrained platforms such as UAVs and embedded systems requires efficient model architectures and hardware-aware optimization strategies. Several studies have evaluated YOLO-based detectors on the NVIDIA Jetson Nano platform. Wadhwa et al. implemented an aerial image detection system and analyzed throughput limitations [24]. Tadjine et al. conducted benchmarking experiments on the LOCO dataset using Jetson Nano, providing insights into dataset partitioning and real-time inference performance [25]. Furthermore, Kurniawan et al. developed a vehicle speed estimation system for UAV video streams based on YOLOv8 deployed on Jetson Nano, achieving near real-time processing capability [26]. Beyond terrestrial scenarios, Iyengar et al. demonstrated an embedded AI pipeline for underwater object recognition, validating the feasibility of real-time forensic analysis in underwater environments [27]. Together, these studies highlight the increasing research emphasis on balancing detection accuracy and computational efficiency for practical edge deployment.

## 3. Materials and Methods

### 3.1. Overall Algorithm Framework

**Figure 1:**
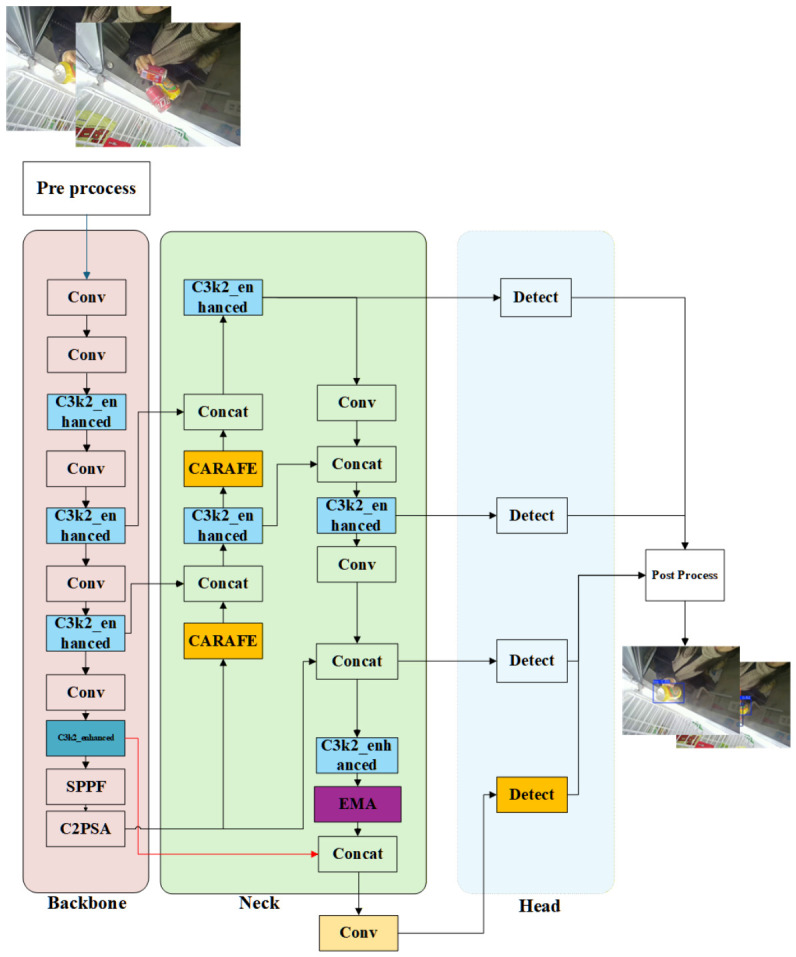
Overall algorithm flowchart.

Figure 1 illustrates the overall architecture of the proposed improved YOLOv26 detection framework. Given an input image $I\in R^{H\times W\times 3}$, the network first extracts hierarchical feature representations through the backbone network. The backbone consists of multiple convolutional layers and C3k2-enhanced modules, which progressively encode spatial and semantic information from low-level features to high-level representations.

Subsequently, the neck module performs multi-scale feature aggregation through a series of upsampling, downsampling, and feature fusion operations.

Following the standard YOLO detection paradigm, the fused feature maps are forwarded to the detection head, which produces prediction results at three feature levels (P3, P4, and P5), corresponding to small-, medium-, and large-scale objects, respectively.

**Figure 2:**
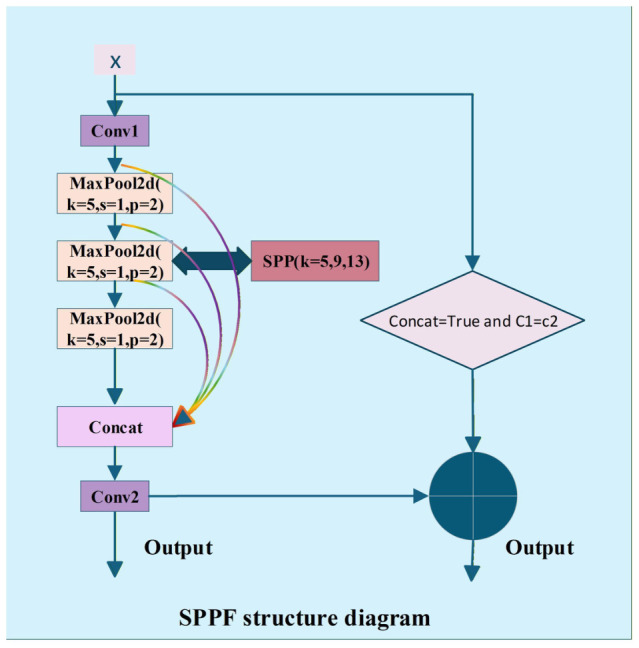
FuseModule Multi-channel fusion module.

As shown in Figure 2, the SPPF module incorporates shortcut connections to facilitate gradient propagation during backpropagation. This design improves training stability and optimization efficiency while preserving computational efficiency.

Overall, the improved YOLOv26 framework enhances feature extraction, multi-scale fusion, and attention modeling while maintaining the lightweight characteristics required for real-time edge deployment.

### 3.2. C3k2-Enhanced Module

**Figure 3:**
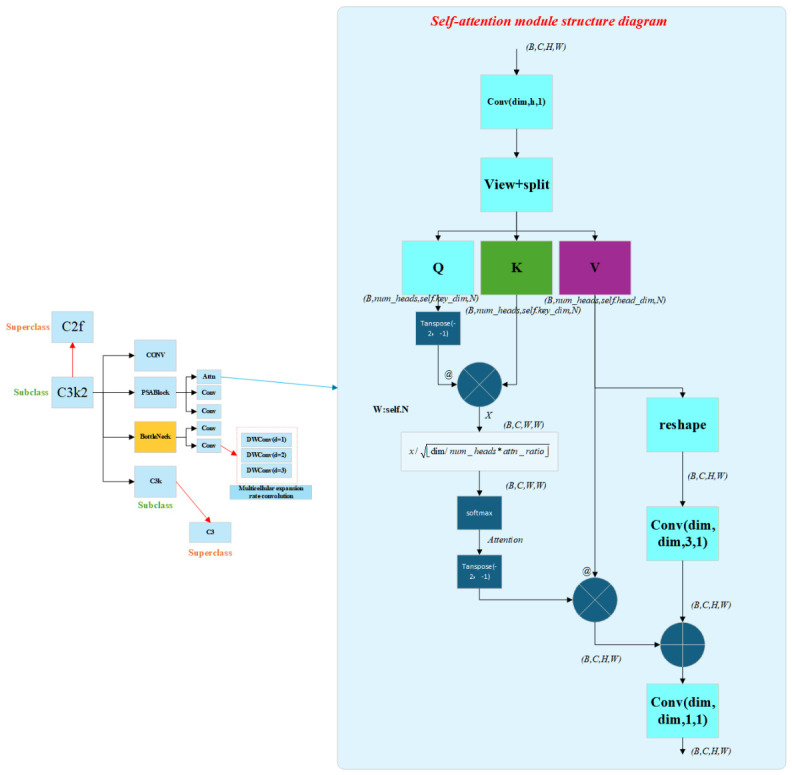
Topology diagram of the enhanced C3k2 module.

A structural inspection of the C3k2 module in YOLOv11 indicates that its original design does not incorporate explicit attention mechanisms such as the PSAModule. The PSAModule follows the classical self-attention paradigm widely adopted in Transformers: query (Q) and key (K) projections are generated from the input feature map to compute an attention matrix, which is subsequently applied to the value (V) representation via weighted aggregation to produce an enhanced feature map. This operation assigns higher weights to informative regions while suppressing background responses, enabling the detector to focus on discriminative target-related cues in cluttered retail scenarios. The algorithmic pipeline of the self-attention mechanism is illustrated in Figure 3.

Further analysis reveals that multiple subcomponents associated with C3k2 are built on bottleneck blocks. Such blocks can be stacked n times to perform repeated feature extraction while preserving the spatial resolution of the input feature maps. However, the receptive field is typically dominated by a single kernel scale, which may be insufficient for complex snack-cabinet environments featuring strong occlusion, large scale variation, and visually cluttered backgrounds. To address this limitation, we introduce dilated convolutions by incorporating the dilation parameter into the convolutional layers. By using different dilation rates, the convolution can capture multi-scale contextual information, effectively enlarging the receptive field without substantially increasing the number of parameters, thereby improving feature representation.

The relationship between the dilation rate and the effective kernel size is summarized in Equation (1). When *d* = 1, the operation degenerates to standard convolution; when using multiple dilation settings (e.g., *d* = 1, 2, 3 in this work), different effective kernel sizes and receptive fields are obtained, leading to richer multi-scale feature extraction.

The effective kernel size of dilated convolution can be expressed as:

(1) $$k_{\mathrm{dilated}}=k+\left(k-1\right)\cdot \left(d-1\right)$$

where *k* is the original convolution kernel size, and *d* denotes the dilation factor. The term $k_{\mathrm{dilated}}$ represents the equivalent receptive field size of the dilated convolution.

A further analysis of the output feature maps reveals the relationship governing spatial dimensional variations, as expressed in Equation (2). In this study, Depthwise Convolution (DWConv) is employed as the feature extraction operator. However, its implementation inherits from the parent Conv class; therefore, the padding parameter (p) is dynamically computed to ensure that the spatial dimensions of the output feature map remain consistent with those of the input. This design enables multi-layer stacked feature extraction while avoiding unnecessary changes in feature map resolution, thereby preserving scale consistency within the network architecture.

To maintain consistent spatial resolution during dilated convolution, the padding value is calculated as:

(2) $$p=\frac{(k-1)\times d}{2}$$

where *p* represents the padding size, *k* denotes the original convolution kernel size, and *d* is the dilation rate. Based on the padding configuration, the output spatial dimension of the feature map can be computed as:

(3) $$o=\left\lfloor \frac{i+2p-k-(k-1)(d-1)}{s}\right\rfloor$$

where *o* is the output feature map size, *i* denotes the input feature map size, and *s* represents the convolution stride.

From the derivation of Equation (3), it can be observed that with appropriately configured stride and padding strategies, the spatial resolution of the output feature map can be preserved. This property is particularly important for multi-scale feature fusion, as it guarantees feature alignment across different branches, ultimately improving fusion effectiveness and training stability.

Finally, the multi-scale feature maps obtained from convolutions with different dilation rates are concatenated along the channel dimension and subsequently passed through convolutional layers for feature fusion and reconstruction. While preserving the spatial dimensional consistency of the original C3k module, this design enables effective integration of multi-scale receptive field information. The proposed structure enhances the model’s capability to represent small objects and complex background patterns without introducing significant computational overhead. Experimental results demonstrate that the modification not only improves the Recall metric but also yields an overall increase in mAP, thereby validating the effectiveness of the multi-dilation-rate feature fusion strategy.

### 3.3. CARAFE Upsampling Module

To improve feature recovery capability during upsampling and alleviate information loss caused by conventional interpolation methods, the Content-Aware ReAssembly of FEatures (CARAFE) [28] module is introduced into the neck structure. Unlike traditional upsampling operators such as nearest-neighbor interpolation, bilinear interpolation, or transposed convolution, CARAFE reconstructs feature maps through content-aware dynamic kernels, enabling more accurate spatial detail recovery.

Specifically, CARAFE consists of two stages: kernel prediction and feature reassembly. In the kernel prediction stage, the input feature map first passes through a channel compression layer and a content encoder to generate adaptive reassembly kernels for each spatial position. These kernels are dynamically generated according to local semantic information rather than fixed convolution weights.

Given an input feature map $X\in R^{C\times H\times W}$, the content-aware reassembly kernel can be expressed as

(4) K=ϕ(X)

where ϕ(·) denotes the kernel generation function. Then, the upsampled feature map is reconstructed through feature reassembly as

(5) $$Y\left(p^{\prime }\right)=\sum_{q\in N(p)}K_{p}\left(q\right)\cdot X\left(q\right)$$

where $Y(p^{\prime })$ represents the reconstructed feature at location $p^{\prime }$, N(p) denotes the receptive field centered at position *p*, and $K_{p}\left(q\right)$ is the adaptive reassembly weight corresponding to position *q*.

Compared with conventional upsampling operators, CARAFE has three main advantages:

- **Large receptive field:** By aggregating contextual information within a larger neighborhood, CARAFE captures richer semantic dependencies than local interpolation methods.
- **Content-aware reassembly:** Dynamic kernels are generated conditioned on input features, enabling adaptive upsampling for different spatial structures and object boundaries.
- **Lightweight and efficient:** CARAFE introduces limited computational overhead while significantly improving feature reconstruction capability.

By integrating CARAFE into the feature fusion stage, the proposed network enhances multi-scale feature representation and improves detection performance, particularly for small objects and occluded targets.

### 3.4. EMA Module

Inspired by the efficient multi-scale attention mechanism proposed by Ouyang et al. [29], this paper introduces an Efficient Multi-scale Attention (EMA) module to enhance feature representation. This module adopts cross-spatial learning and lightweight operations to achieve efficient attention modeling, which effectively captures multi-scale contextual information while maintaining low computational overhead. By focusing on discriminative regions and suppressing irrelevant background noise, the EMA module significantly improves the detection performance for small targets under low-light conditions without introducing excessive computation burden.

**Figure 4:**
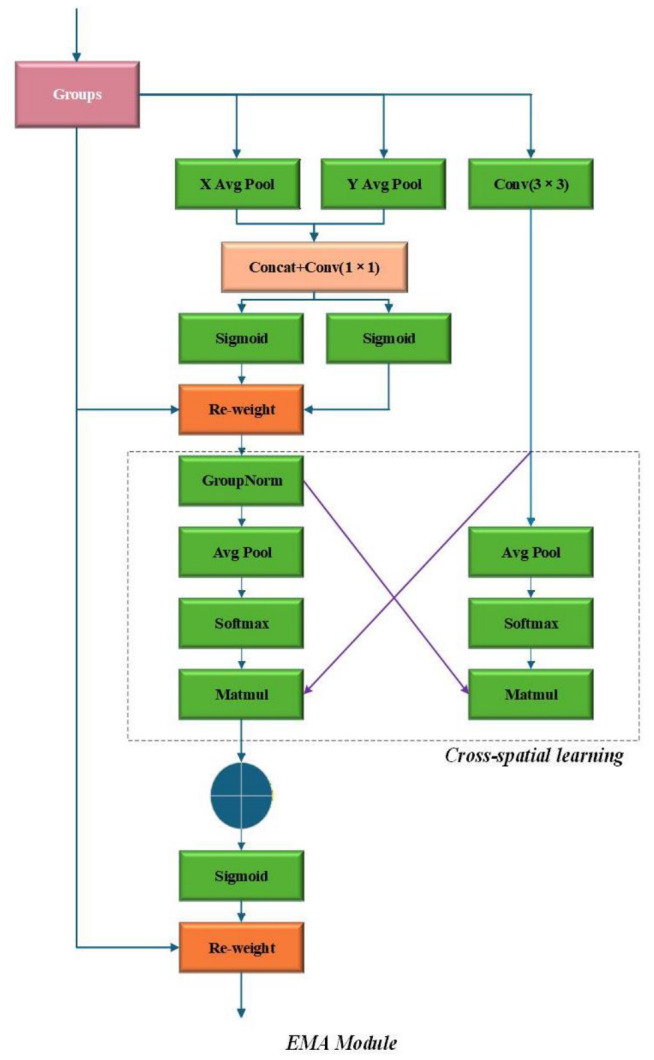
EMA Attention Mechanism.

The structure of the EMA module is illustrated in Figure 4. The module utilizes spatial average pooling and depthwise separable convolution, combined with cross-spatial pathways, to construct a salient feature representation mechanism. This design enhances the responses of important regions while simplifying the attention computation process, thereby improving the efficiency of semantic information modeling. By focusing on key feature information across different dimensions, the EMA module strengthens the feature representation capability of the backbone network. Moreover, the dual-branch design of the EMA module facilitates more effective feature interaction, which contributes to improved performance in multi-class object recognition tasks.

Assume that the input feature map is defined as

(6) $$X\in R^{C\times H\times W}$$

where *C*, *H*, and *W* denote the number of channels, height, and width of the feature map, respectively.

EMA first divides the input channels into *G* groups:

(7) $$X=\{X_{1},X_{2},\ldots ,X_{G}\}$$

where

(8) $$X_{i}\in R^{\frac{C}{G}\times H\times W},\;i=1,2,\ldots ,G$$

This grouping strategy reduces computational complexity while enhancing cross-channel feature interaction.

The overall input–output relationship of the EMA module can be expressed as

(9) $$Y=X⊗\sigma \left(Conv\left(\delta \left(Conv(\left[P_{h}\left(X\right),P_{w}\left(X\right)\right])\right)\right)\right)$$

where $P_{h}\left(\cdot \right)$ and $P_{w}\left(\cdot \right)$ represent horizontal pooling and vertical pooling operations, respectively. The operators [·], Conv(·), δ(·), and σ(·) denote concatenation, convolution, ReLU activation, and Sigmoid activation functions, respectively, while ⊗ represents element-wise multiplication.

Through such fine-grained operations at the channel level, the EMA module enables the network to effectively capture discriminative features, thereby improving the model’s ability to learn critical feature representations.

## 4. Results and Analysis

### 4.1. Experimental Environment and Dataset Construction

As shown in Table 1, the experimental hardware and software configurations in this study are presented in detail. The ablation experiments are conducted on a desktop computer located in the East Campus of Yangtze University, Jingzhou District, Jingzhou City, Hubei Province, China, running the Windows operating system. For generalization verification, experiments are deployed on a high-performance Ubuntu server rented from AutoDL (https://www.autodl.com/market/list, (accessed on 1 January 2026)). To ensure the rationality and comparability of experimental results, all experiments are carried out under strict control of variables, with the ablation studies implemented incrementally by modifying individual components. A unified deep learning framework and consistent training hyperparameters (including learning rate, batch size, and training epochs) are adopted across all experiments, effectively eliminating external interference and guaranteeing the consistency of training conditions.

In this study, a self-built dataset was constructed using a fisheye camera to capture video streams in an intelligent retail cabinet scenario. The collected videos were subsequently segmented into image frames to form the dataset. The dataset contains common retail commodities typically found in daily vending cabinets, with a total of 6467 images.

All images were manually annotated using the *LabelImg* annotation tool and converted into the YOLO annotation format. The dataset was divided into training, validation, and test sets according to the ratio of 8:1:1. Specifically, the training set contains 5167 images, the validation set contains 638 images, and the test set contains 662 images.

The self-built dataset includes 20 commodity categories, which can be categorized into several major packaging types, including bottled products, canned products, bagged goods, and boxed commodities. These categories cover the majority of products commonly found in retail vending cabinets, enabling the model to learn representative features under realistic retail scenarios.

**Figure 5:**
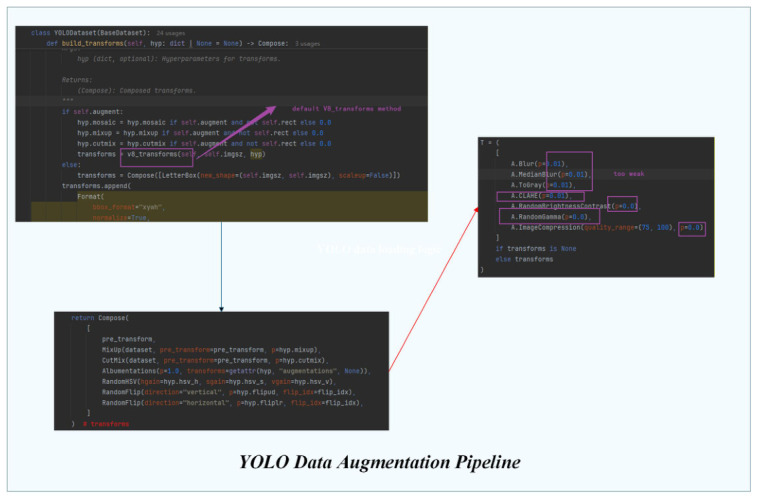
YOLO Data Augmentation Pipeline.

The overall data augmentation pipeline adopted in this study is illustrated in Figure 5. During the training stage, the YOLO data loader constructs the transformation pipeline through a composed structure that integrates multiple augmentation operations. The pipeline includes commonly used techniques such as MixUp, CutMix, HSV color-space perturbation, random flipping, and Albumentations-based image transformations.

Furthermore, several illumination-related operations, including contrast enhancement, gamma correction, color perturbation, and noise injection, are introduced to simulate diverse lighting conditions commonly encountered in retail cabinet environments. These transformations increase the diversity of the training data and improve the robustness of the detection model under varying illumination conditions.

To further evaluate the generalization capability of the proposed model, several public datasets were also introduced for supplementary experiments. One of them is the commodity dataset released in the Sixth Xinye Technology Cup (https://ai.ppdai.com/mirror/goToMirrorDetailSix?mirrorId=26 (accessed on 1 January 2026)) Image Algorithm Competition, which contains a total of 5422 annotated images covering 20 commodity categories. In addition, the large-scale Retail Product Checkout (RPC) dataset [30] released by Megvii Technology was also employed for further validation. These third-party datasets are used to evaluate the robustness and generalization capability of the proposed model.

Regarding the experimental environment, model training was conducted on two different computing platforms. The left configuration corresponds to a personal workstation, while the right configuration corresponds to a cloud server environment. Since hardware differences may influence training efficiency and convergence behavior, all experiments were performed following the principle of controlled variables to ensure fair comparisons between models.

### 4.2. Dataset Construction and Augmentation

In this study, video data were collected using a fisheye camera, and image frames were extracted and processed to construct a self-built dataset. The dataset consists of common items typically found in daily retail cabinets. As shown in Table 2, a total of 6467 images were collected and manually annotated in YOLO format using the LabelImg tool. In this work, image annotation is performed using the LabelImg tool (https://github.com/tzutalin/labelImg (accessed on 1 January 2026)).

To improve the robustness of the model and alleviate the limitation of the relatively small dataset size, data augmentation techniques were applied to the training set. Specifically, brightness adjustment, contrast enhancement, and gamma correction were employed to simulate different illumination conditions. Through these augmentation operations, the number of training samples increased from 5167 images to 25,835 images, which is approximately five times the original training set size. This significantly increased the diversity of the training data and enhanced the generalization capability of the proposed model.

To further evaluate the generalization ability of the proposed method, additional public datasets were incorporated. A dataset from the Sixth Xinye Technology Cup Image Algorithm Competition was adopted, which contains 5422 annotated images across 113 commodity categories. In addition, the large-scale RPC dataset released by Kuangshi Technology was included for further evaluation. Subsequent experiments validate the effectiveness and generalization performance of the proposed model on these third-party datasets.

Furthermore, the experiments were conducted on two different computational platforms: a personal PC and a cloud server. Since training performance may vary across different hardware environments, all comparative experiments were performed under the controlled variable principle to ensure fairness and reliability in performance evaluation.

**Figure 6:**
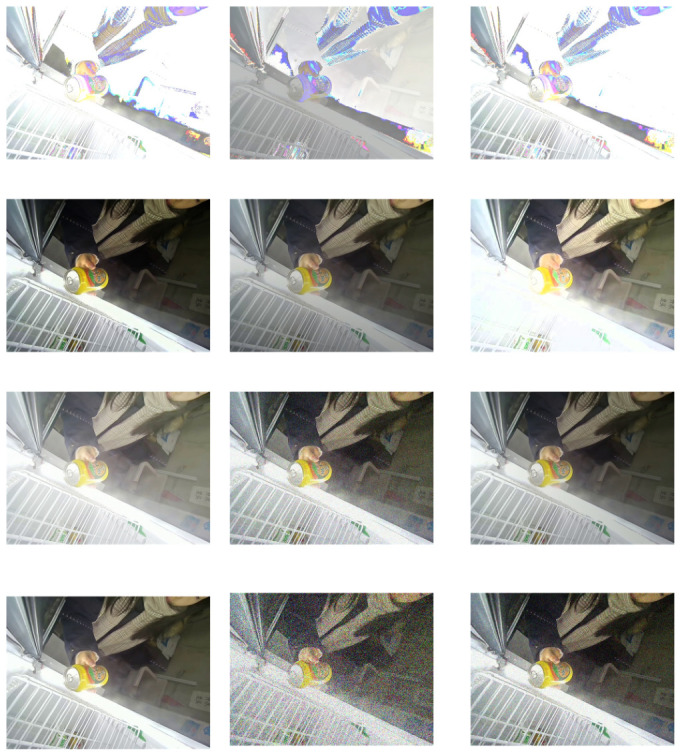
Different light.

As shown in Figure 6, to improve the robustness of the detection model under complex lighting conditions, several illumination augmentation techniques are applied during data preprocessing. These methods simulate real-world lighting variations, including brightness changes, contrast adjustments, gamma transformation, illumination noise, and shadow generation. Figure shows the visual effects of different illumination augmentation operations. From top to bottom, the rows correspond to brightness adjustment, contrast enhancement, gamma correction, and noise/shadow simulation. In each row, the first column is the original image, while the subsequent columns display the augmented results under different parameter settings, including brightness scaling, contrast stretching, gamma transformation, and noise/shadow injection. These operations simulate diverse illumination conditions encountered in retail cabinet environments, thereby improving the model’s robustness to varying lighting scenarios.

**Brightness Adjustment.** This operation modifies the brightness channel in the HSV color space to achieve linear brightness variation while avoiding color distortion caused by direct manipulation in the RGB space. The transformation can be expressed as

(10) $$V_{\mathrm{new}}=\mathrm{clip}\left(V_{\mathrm{old}}\times \alpha ,0,255\right),$$

where α is the brightness scaling factor. When α>1, the image brightness increases, whereas α<1 results in a darker image.

**Contrast Enhancement.** Contrast enhancement stretches the distribution of pixel intensities through a linear transformation, thereby emphasizing the grayscale differences between different regions and highlighting target details. The transformation is defined as

(11) $$I_{\mathrm{new}}=\mathrm{clip}\left((I_{\mathrm{old}}-128)\times \beta +128,0,255\right),$$

where β represents the contrast adjustment factor. A value of β>1 increases contrast, while β<1 reduces contrast.

**Gamma Correction.** Gamma correction applies a power-law transformation to simulate the nonlinear perception of illumination by the human visual system. It is commonly used to correct overexposed or underexposed images. The transformation is expressed as

(12) $$I_{\mathrm{new}}=255{\left(\frac{I_{\mathrm{old}}}{255}\right)}^{\gamma },$$

where γ<1 brightens the image (simulating overexposure), whereas γ>1 darkens the image (simulating insufficient illumination).

**Light Noise Simulation.** To mimic uneven illumination and sensor noise in real-world environments, Gaussian noise is added to the image. This strategy enhances the robustness of the model to noisy lighting conditions. The process can be formulated as

(13) $$I_{\mathrm{noisy}}=\mathrm{clip}(I_{\mathrm{clean}}+N\left(0,\sigma^{2}\right),0,255),$$

where $N(0,\sigma^{2})$ denotes Gaussian noise with mean 0 and variance $\sigma^{2}$.

**Shadow Generation.** To simulate directional illumination occlusion, a linear gradient shadow mask is generated and multiplied with the original image to produce realistic shadow effects. The transformation can be expressed as

(14) $$I_{\mathrm{shadow}}=I_{\mathrm{original}}\times M,$$

where *M* represents the generated shadow mask.

### 4.3. Evaluation Indicators

(1) AP [31]

Given a total number of classes *n*, let $r_{k}$ denote the recall value at the *k*-th threshold, and $p_{k}$ represent the corresponding maximum precision when the recall is greater than or equal to $r_{k}$. The average precision (AP) is defined as:

(15) $$\mathrm{AP}=\sum_{k=1}^{n}p_{k}\left(r_{k+1}-r_{k}\right).$$

(2) Recall [10]

(16) Recall=TPTP+FN

In the formula, *FN* represents the total number of false negatives and TP denotes the number of true positives.

(3) Precision [31]

Precision represents the proportion of true positive samples detected by the algorithm relative to the total number of positive samples detected, defined as follows.

(17) $$\mathrm{Pr}ecision=\frac{TP}{TP+FP},$$

where TP denotes true positives and FP denotes false positives.

(4) mAP [28]

The mean average precision (mAP) is defined as the average of the average precision (AP) across all categories, formulated as follows:

(18) $$mAP=\frac{1}{N}\sum_{i=1}^{N}AP_{i},$$

where N denotes the total number of categories and $AP_{i}$ represents the average precision for the *i*-th category.

(5) F1 [32]

To validate the robustness of the model, this study uses the F1-score, which is the harmonic mean of precision and recall, formulated as follows.

(19) F1=2Precise·RecallPrecise+Recall=2TP2TP+FP+FN.

(6) Inference Time [33]

(20) $$T_{\inf}=T_{\mathrm{pre}}+T_{\mathrm{model}}+T_{\mathrm{post}}$$

The total inference time consists of three components: preprocessing time, model forward inference time, and post-processing time, which jointly determine the real-time performance of the object detection framework and are crucial for practical application scenarios such as intelligent retail cabinet commodity detection. Specifically, the preprocessing stage is a critical prerequisite for ensuring the stability and accuracy of model inference, involving a series of sequential and refined operations to convert raw input images into standardized feature tensors that can be directly processed by the neural network. First, the input images collected from the retail cabinet are resized to the fixed input resolution required by the YOLO26 model (e.g., 640 × 640 pixels), using interpolation methods (such as bilinear interpolation) to avoid image distortion and ensure the integrity of commodity feature information. Subsequently, pixel value normalization is performed to scale the pixel intensity of the image from the original 0–255 range to the 0–1 range, which helps accelerate the convergence of the neural network and reduce the impact of numerical differences on model training and inference. In addition, channel dimension adjustment is carried out to align the image channel order with the input requirements of the model (e.g., converting the RGB channel order to the BGR order required by the PyTorch framework), and finally, the processed image is converted into a tensor format, which is the standard data type for neural network computation. These preprocessing operations are lightweight but essential, laying a solid foundation for subsequent efficient and accurate model inference.

The model inference time corresponds to the forward propagation process of the YOLO26 neural network, during which the standardized feature tensors are input into the network, and multi-scale feature extraction, feature fusion, and detection prediction are completed through multiple convolutional layers, attention modules, and prediction heads, ultimately outputting the preliminary detection results including bounding box coordinates, category classification probabilities, and confidence scores.

The post-processing stage is where YOLO26 differs significantly from other object detection models (including traditional YOLO series models such as YOLOv3, YOLOv4, YOLOv5, and even the latest YOLOv8, as well as other mainstream detection frameworks like Faster R-CNN, SSD, and RetinaNet). For most of these conventional models, non-maximum suppression (NMS) is an indispensable post-processing step: due to the overlapping of detection frames predicted by the model for the same target, NMS is required to screen out the bounding box with the highest confidence score, suppress redundant and overlapping detection frames, and avoid multiple detections of the same target, thereby ensuring the rationality of the final detection results. However, the proposed YOLO26 model innovatively optimizes the prediction head structure and loss function, introducing an adaptive frame selection mechanism that enables the model to directly output non-overlapping, high-precision bounding boxes during forward inference. This design completely eliminates the need for the NMS operation in the post-processing stage, simplifying the post-processing procedure significantly. The post-processing of YOLO26 only includes lightweight operations: decoding the predicted bounding box coordinates (converting the normalized coordinates output by the model into actual pixel coordinates corresponding to the original image), analyzing the category classification probability to determine the category of each detected commodity, and filtering out detection results with confidence scores lower than the set threshold (e.g., 0.5) to remove false positive detections.

Compared with conventional models that require NMS, the elimination of the NMS operation in YOLO26 not only reduces the additional computational overhead caused by NMS sorting and screening but also avoids the problem of missed detections or false suppressions caused by improper NMS parameter settings (such as the intersection-over-union (IoU) threshold). As a comprehensive evaluation metric, the total inference time covers the entire execution process of the detection pipeline, objectively reflecting the real-time performance and computational efficiency of the YOLO26 object detection framework, which is particularly suitable for real-time detection scenarios with high efficiency requirements such as intelligent retail cabinets.

### 4.4. Baseline Model Comparison Experiments

YOLOv26 was selected as the baseline model after comprehensively considering both detection accuracy and real-time performance. To ensure fairness and reproducibility, several representative state-of-the-art (SOTA) detectors were evaluated under unified experimental settings on the UAV-based object detection benchmark, VisDrone2019-DET. VisDrone2019-DET is characterized by a high proportion of small objects, densely distributed targets, and frequent occlusions, making it well-suited for evaluating multi-scale feature representation and localization robustness under complex aerial perspectives. The dataset contains 6471 training images, 548 validation images, and 1610 test images, covering 10 common categories of road traffic participants and pedestrians. The comparative results are summarized in Table 3.

As shown in Table 3, on the VisDrone2019-DET benchmark, different models exhibit noticeable variations in both detection accuracy and real-time performance. YOLOv8n achieves the highest mAP@50 (0.313); however, this comes with a relatively higher computational complexity. In contrast, YOLOv26 requires only 5.2 GFLOPs and achieves an inference time of 0.7 ms, demonstrating strong real-time capability while maintaining a competitive mAP@50 of 0.297, which is comparable to other mainstream lightweight detectors. The proposed improved model further increases the recall to 0.325 with almost no additional computational overhead, indicating a more favorable balance between accuracy and efficiency. Based on this trade-off analysis, YOLOv26 is selected as the baseline model for subsequent improvements.

Subsequent comparative performance analysis of various baseline models in our custom dataset reveals detailed results presented in Table 3.

### 4.5. Comparative Experiments on the Self-Constructed Dataset

As shown in Table 4, on the self-constructed dataset, all models achieve relatively high overall accuracy. YOLOv8n attains the highest mAP50 value (0.977), but with comparatively higher computational cost. YOLOv26 requires only 5.2 GFLOPs and achieves an inference time of 1.5 ms, while maintaining a lightweight advantage with an mAP50 of 0.974 and an mAP50–95 of 0.636. Under the same inference time, the improved model further increases Recall to 0.962, demonstrating enhanced detection stability and overall performance, thereby validating the effectiveness of the proposed approach.

### 4.6. Heatmap Visualization Experiments

**Figure 7:**
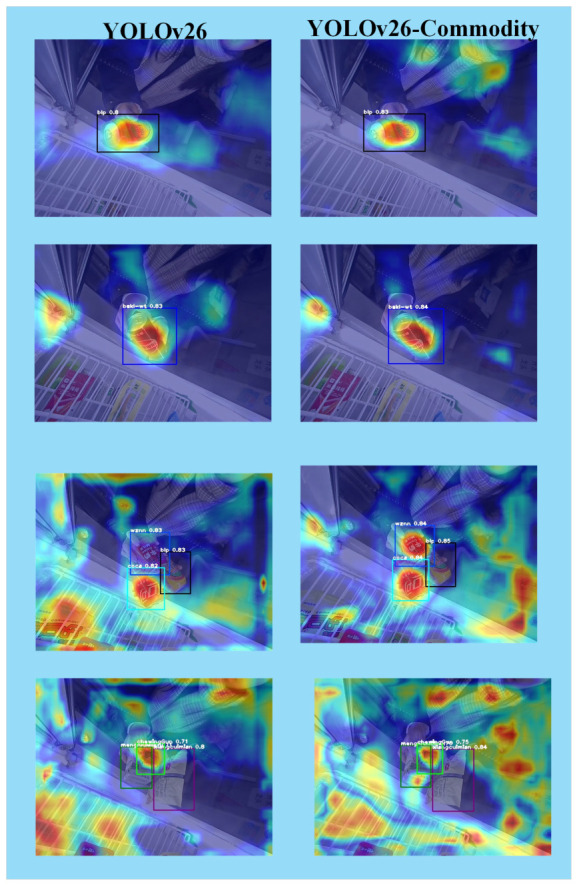
Thermal visualization under the attention model.

As illustrated in Figure 7, the heatmaps present the visualization results of YOLOv26-Commodity under different levels of occlusion, along with the corresponding detection accuracy. In addition, a comparative visualization analysis is conducted between models with and without the attention mechanism to evaluate their respective detection behaviors. By integrating Grad-CAM into YOLOv26, heatmap-based visualization effectively highlights the regions of interest attended by the model, thereby providing intuitive insights into its decision-making process and detection accuracy.

As shown in Figure 7, the heatmaps compare the feature response maps of the baseline YOLOv26 model and the proposed improved model. The first column shows the detection results of the original model, while the second column presents those of the improved model. It can be clearly observed that the proposed model outperforms the baseline in detection accuracy and robustness under various complex retail cabinet scenarios.

In the single-object case (top row), both models successfully activate the target commodity, but the improved model exhibits more concentrated and precise feature responses within the bounding box, with stronger activation intensity and less background interference. In the multi-object dense scenario (middle row), the baseline model shows scattered and overlapping responses, making it prone to confusion between adjacent commodities. In contrast, the improved model clearly distinguishes each target with independent, well-defined activation regions, effectively suppressing interference from similar-looking background elements. In the occluded and small-object case (bottom row), the baseline model’s response to partially blocked or small-sized commodities is significantly weakened, leading to low confidence scores and potential missed detections. The improved model, however, maintains stable and high-confidence activation even under occlusion, demonstrating stronger feature extraction capability for challenging targets.

From the heatmaps, red regions indicate areas receiving high attention from the model. The proposed model, enhanced with the attention mechanism, focuses more precisely on the discriminative visual features of commodities such as logos, text, and packaging patterns, rather than being distracted by irrelevant background noise. This targeted feature enhancement results in more concentrated color distribution and clearer hierarchical structures in the activation maps, indicating improved region selection capability and feature representation.

Overall, the visualization results confirm that the improved model achieves more stable and accurate detection performance across different scenarios, including single-object, multi-object dense, and occluded cases. The enhanced attention mechanism effectively guides the model to focus on key commodity features, thereby improving overall detection accuracy and robustness in complex retail cabinet environments.

### 4.7. Attention Mechanism Comparison Experiments

To further validate the effectiveness of the introduced modules, attention mechanism comparison experiments were conducted based on the final model configuration, in which the regression loss function was set to Complete Intersection over Union (CIoU). The experiments were performed in a deep learning environment equipped with an NVIDIA GeForce 4070 Ti GPU (16,380 MiB memory), with the number of epochs set to 100 and the number of workers set to 8.

As shown in Table 5, The comparative analysis of attention mechanisms reveals that the proposed Efficient Multi-scale Attention (EMA) module achieves a superior balance between detection accuracy and computational efficiency, outperforming existing methods in both precision and stability. As shown in Table 4, Efficient Multi-scale Attention (EMA) attains the highest mean Average Precision at IoU = 0.5 (mAP@0.5) of 0.976, a 0.2% improvement over the baseline (NO), while maintaining a competitive Recall of 0.953. Notably, this performance gain is achieved without incurring additional computational overhead: Efficient Multi-scale Attention (EMA) shares the lowest Giga Floating-point Operations Per Second (GFLOPs) of 5.7 with Efficient Channel Attention (ECA) and maintains an inference time of 1.8 ms, matching the efficiency of lightweight alternatives like Efficient Channel Attention (ECA) and Squeeze-and-Excitation Network (SENet), and outperforming Convolutional Block Attention Module (CBAM) and Coordinate Attention (CA) by 0.2 ms. This stability stems from Efficient Multi-scale Attention (EMA)’s streamlined design, which avoids the redundant computations inherent in multi-branch attention structures (e.g., Convolutional Block Attention Module (CBAM)’s channel and spatial branches). By focusing on critical feature refinement without excessive complexity, Efficient Multi-scale Attention (EMA) effectively mitigates the performance fluctuations observed in other methods—such as the trade-off between Recall and mean Average Precision at IoU = 0.5 (mAP@0.5) in Convolutional Block Attention Module (CBAM), or the slight mean Average Precision at IoU = 0.5 (mAP@0.5) drop in Squeeze-and-Excitation Network (SENet). The consistent performance across all metrics, coupled with its low computational footprint, positions Efficient Multi-scale Attention (EMA) as a robust and practical solution for real-time deployment on edge devices like Jetson Nano, where both accuracy and latency are critical constraints. In summary, Efficient Multi-scale Attention (EMA) not only enhances detection precision but also preserves the model’s efficiency, demonstrating a more stable and reliable performance profile compared to conventional attention mechanisms. This makes it particularly well-suited for challenging retail scenarios, where the need for accurate, real-time commodity recognition must be balanced against the computational limitations of embedded platforms.

### 4.8. Multi-Module Fusion Comparison Experiments

Table 6 presents multi-module ablation experiments to evaluate the individual and combined contributions of the proposed C3k2-enhanced module, CARAFE upsampling operator, and EMA + CONV attention mechanism, as well as their impact on both detection performance and computational complexity (measured in GFLOPs). The baseline model (Method 1), which incorporates none of the three modules, achieves an mAP50 of 0.974 and a Recall of 0.945 with 5.8 GFLOPs, serving as the reference for subsequent comparisons. Introducing the C3k2-enhanced module and CARAFE operator together (Method 2) results in a modest increase in computational overhead, with GFLOPs rising from 5.8 to 6.0 (a 3.4% increase). In exchange for this slight computational cost, consistent performance improvements are observed across key metrics: mAP50 increases to 0.976, and Recall rises to 0.952. This gain stems from the complementary roles of the two modules: the C3k2-enhanced module strengthens multi-scale feature aggregation to enrich both low-level spatial details and high-level semantic representations, while the CARAFE operator improves upsampling spatial precision to reduce feature blurring during multi-scale fusion. Together, they lay a foundation for enhanced detection performance with only a minor computational trade-off.

In contrast, applying the CARAFE module alone (Method 3) leads to a more significant increase in computational overhead (GFLOPs = 6.2, a 6.9% increase from the baseline) but a marginal decline in detection performance, with mAP50 dropping to 0.971 and Recall falling to 0.934. This counterintuitive result indicates that the benefits of content-aware reassembly in CARAFE cannot be fully realized without complementary structural enhancements. Without the strengthened feature representations provided by the C3k2-enhanced module, CARAFE’s refined upsampling fails to deliver meaningful performance gains, and the additional computational cost even introduces minor feature alignment errors that degrade detection accuracy.

The full integration of all three modules (C3k2-enhanced + CARAFE + EMA + CONV, Method 4) yields the best overall detection performance, achieving the highest Recall of 0.954 while maintaining an mAP50 of 0.976. However, this configuration requires the highest computational overhead in the experiments, with GFLOPs reaching 6.3 (an 8.6% increase from the baseline). This performance improvement is thus achieved at the cost of a moderate increase in model complexity and computational burden. The EMA + CONV attention mechanism plays a critical role in this trade-off: it refines channel-wise feature responses to emphasize discriminative commodity features, mitigating the limitations of standalone modules and amplifying the benefits of both the enhanced multi-scale features from C3k2 and the precise upsampling from CARAFE. As a result, the model’s ability to detect challenging cases (such as small or partially occluded commodities) is significantly improved, even as the total computational budget increases.

Overall, the ablation experiments demonstrate that the proposed module combination delivers clear performance gains, particularly in Recall, but these improvements are accompanied by a gradual increase in model complexity and computational overhead as more modules are introduced. While the synergistic integration of all three modules achieves a favorable balance for practical deployment in terms of detection accuracy, the 8.6% increase in GFLOPs (from 5.8 to 6.3) highlights a key limitation: the enhanced model is more computationally intensive, which may limit its deployment on resource-constrained edge devices such as embedded controllers in intelligent retail cabinets.

Therefore, future work will focus explicitly on reducing model complexity while preserving the performance gains achieved in this study. Planned optimizations include: (1) lightweight redesign of the CARAFE module to simplify its content-aware upsampling pipeline and eliminate redundant computations; (2) structured pruning of the C3k2-enhanced and EMA + CONV modules to remove low-contribution channels and reduce parameter count; (3) model quantization (e.g., INT8 quantization) and knowledge distillation to compress the model without significant accuracy loss; and (4) further refinement of the module combination based on the ablation results, to identify a more compact architecture that maintains high detection performance with lower computational overhead. These efforts aim to make the model more efficient and suitable for real-time deployment in resource-limited retail scenarios.

### 4.9. Generalization and Illumination Comparison Experiments

Since the model proposed in this study is trained on a self-constructed dataset, its local performance is reliable, with satisfactory mAP, Recall, and inference speed. However, as the developed commodity recognition model is intended for deployment across different retail cabinets, additional generalization and illumination experiments were conducted to evaluate its performance on public datasets.

The RPC [30] dataset was adopted for generalization experiments. For categories overlapping with the self-constructed dataset, 1000 images were collected for evaluation. A comparative analysis was performed between YOLOv26 and YOLOv26-Commodity, and the corresponding results are presented as follows.

**Figure 8:**
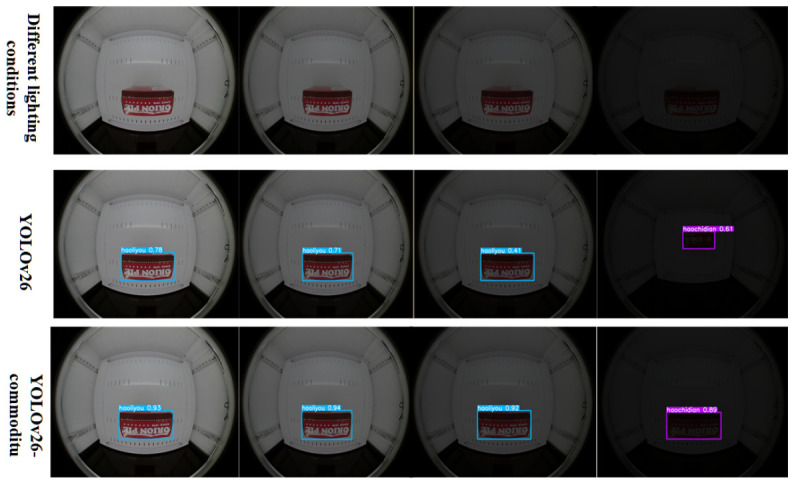
Illumination Comparison1.

**Figure 9:**
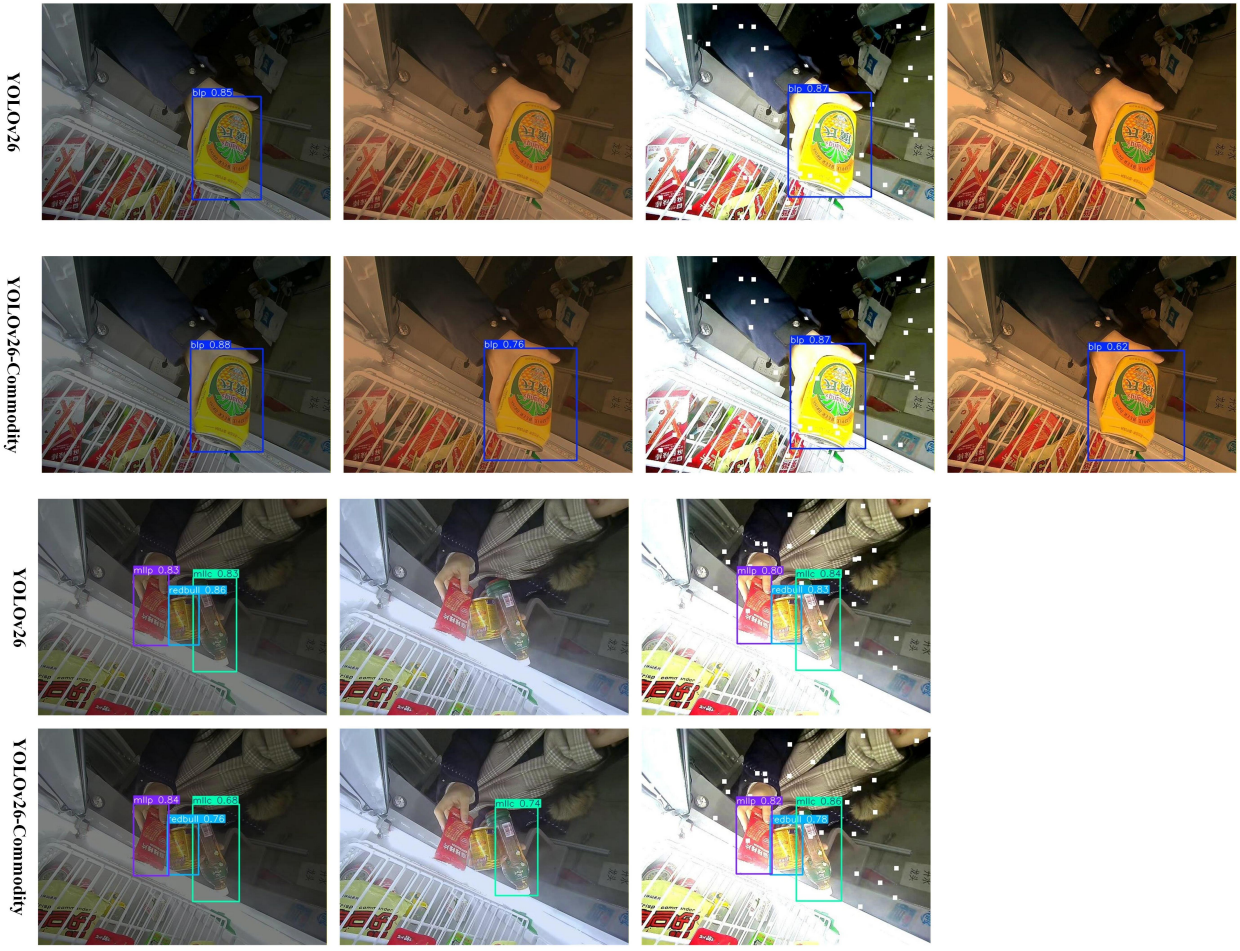
Illumination comparison2.

To further evaluate the illumination robustness of the proposed model, detection results under varying lighting conditions are visualized in Figure 8 and Figure 9.

In Figure 8, the first row presents the raw input images under four sequential illumination levels, with brightness gradually decreasing from left to right, simulating the natural lighting attenuation in a closed intelligent retail cabinet. The second row shows the detection results of the baseline YOLOv26 model, while the third row corresponds to the proposed YOLOv26-Commodity model. In the first three columns with normal or moderately reduced brightness, both models achieve stable detection with high confidence scores. However, in the rightmost extreme low-light scenario, the baseline model exhibits a significant drop in confidence (from 0.78 to 0.51) and a misaligned bounding box, whereas the improved model maintains a clear, accurate bounding box and a relatively high confidence score of 0.83, demonstrating substantially enhanced robustness to low-light conditions.

In Figure 9, we further evaluate the model performance under more complex visual distortion scenarios. The first two rows compare single-commodity detection results under varying illumination and viewing angles. From left to right, the columns correspond to normal lighting, moderate brightness reduction, Gaussian noise injection, and strong specular reflection, which are the most common visual degradation phenomena in practical retail cabinet environments. The improved model maintains precise bounding box localization and stable confidence scores even under strong noise and uneven illumination. The bottom two rows extend the evaluation to multi-commodity dense detection in cluttered backgrounds, showing that the proposed method effectively distinguishes adjacent and partially overlapping commodities, while the baseline model is prone to false detection, missed detection, and bounding box drift.

**Figure 10:**
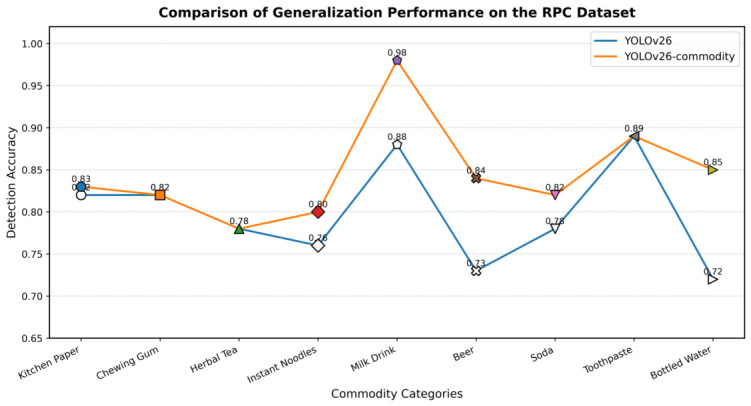
Generalization and comparison experiments on RPC datasets.

**Figure 11:**
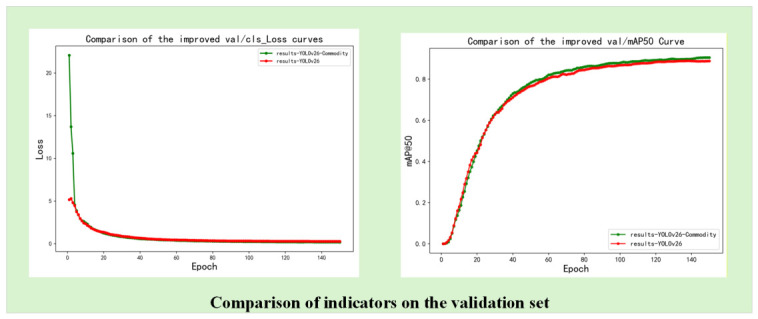
Performance Metrics Analysis of the Xinye Technology Commodity Dataset.

Quantitative generalization performance on public benchmark datasets is further analyzed in Figure 10 and Figure 11. The training and validation curves in Figure 10 demonstrate that the improved model achieves faster convergence and consistently higher mAP@50 than the baseline throughout the entire training process, confirming the effectiveness of the proposed architectural modifications. In Figure 11, detailed category-wise AP values on the Xinye Technology Commodity Dataset are presented, showing that the proposed method achieves consistent accuracy improvements across different commodity categories while maintaining acceptable computational complexity. These results collectively validate that the improved YOLOv26-Commodity model strikes a favorable balance between detection performance and real-time inference efficiency, making it suitable for practical deployment in intelligent retail cabinet scenarios.

### 4.10. AP Comparison Experiments on Public Datasets

To quantitatively analyze the Average Precision (AP) values of different commodities, 100 images were prepared for each product for comparative evaluation. The dataset covers various scenarios, including different lighting conditions and shooting angles. Figure 10 presents partial testing results comparing YOLOv26 and the YOLOv26-Commodity model. The experimental results indicate that the improved model achieves a noticeable improvement in detection accuracy, further validating its effectiveness and precision. The following section presents detailed results on two public benchmark datasets.

As shown in Figure 11, the comparison indicates that the improved model achieves faster convergence on the validation set and maintains a consistently higher mAP@50 compared to the original model. This improvement confirms the effectiveness of the proposed architectural modifications. Although a slight increase in model complexity is observed, the overall balance between detection accuracy and computational cost remains appropriate for practical deployment.

### 4.11. Embedded Device Deployment

**Figure 12:**
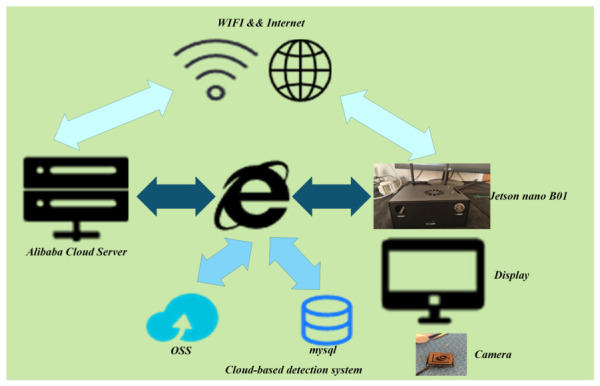
Architecture of the Cloud-Based Detection System.

As shown in Figure 12, this system adopts a cloud–edge collaborative deployment architecture. On the embedded side, a Jetson Nano B01 is utilized as the front-end device, which is connected to a camera and a display to accomplish image acquisition and result visualization. The edge-side software is developed based on Python 3.10 and Tkinter 8.6, implementing functions such as image capture, network communication, and detection result visualization.

The captured images are transmitted via WiFi to the Internet and uploaded to an Alibaba Cloud server, where model inference is performed in the cloud and the detection results are returned to the embedded device. The detection records are stored in a MySQL database for administrative review within the backend management system, while image data are stored in Object Storage Service (OSS).

This architecture effectively balances real-time performance and scalability, facilitating model updates and remote maintenance while ensuring stable system operation.

**Figure 13:**
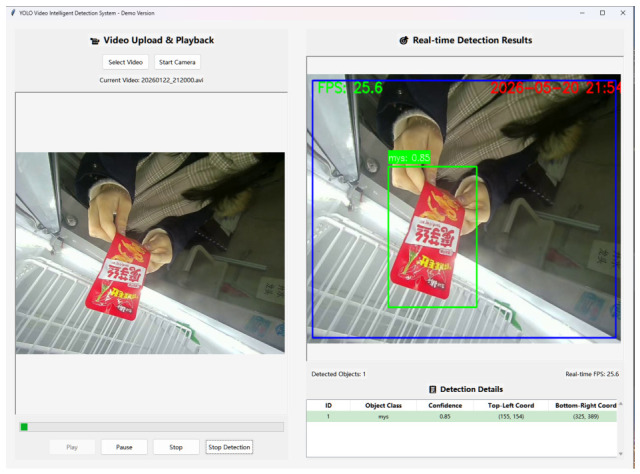
Practical Tkinter Interface Demonstration.

Figure 13 presents the local simulation interface used in practical detection, which supports both real-time image capture and video upload modes for detection. The achieved FPS satisfies practical application requirements. When deployed on the Jetson Nano platform, the overall principle remains the same; however, due to differences in GPU architecture, the model output format must be converted accordingly. In general, the trained weights are first exported to the ONNX format and then converted into an engine file using dedicated tools for inference, which can approximately double the FPS. Therefore, deployment on embedded devices is also feasible in practice.

In future work, this study will further focus on embedded deployment in complex real-world scenarios and explore more efficient and practical solutions.

## 5. Discussion

This study is developed based on the YOLOv26 baseline model, and several targeted structural improvements are introduced to enhance its detection performance in intelligent retail cabinet scenarios, among which the integration of the CARAFE (Content-Aware ReAssembly of FEatures) module is a key optimization. Specifically, the original C3k2 module in YOLOv26 is redesigned as the C3k2-enhanced module, which enhances the network’s feature extraction capability by strengthening the propagation of contextual information, enabling the network to generate richer and more discriminative feature representations and improving its ability to capture complex visual patterns of retail commodities. During the feature fusion stage, on the basis of multi-scale feature aggregation, the Efficient Multi-scale Attention (EMA) module is first incorporated to strengthen the extraction of high-level semantic information, and then the CARAFE module is introduced to realize content-aware feature upsampling and alignment. Unlike traditional bilinear interpolation upsampling, the CARAFE module adaptively assembles local feature patches according to the content of feature maps, effectively solving the problems of feature blurring and detail loss during upsampling, thereby enhancing the alignment and fusion effect of multi-scale features, especially for small-sized commodities in retail cabinets. In addition, an extra Detect head is added to further improve detection accuracy, which, combined with the feature enhancement effect of the CARAFE module, achieves more precise localization and classification of small objects.

To enhance the robustness of the model under varying illumination conditions (such as low light, strong reflection, and uneven lighting) in practical retail scenarios, a series of data augmentation strategies are applied during the training process, including brightness adjustment, gamma correction, noise injection, and shadow simulation. These augmentations effectively increase the diversity of the training data, and the CARAFE module further helps the model to better capture and retain key feature details in augmented images, allowing the model to adapt to different lighting environments more effectively and thereby further improving the overall detection accuracy and generalization ability.

Overall, the proposed modifications (including the C3k2-enhanced module, EMA module, CARAFE module, and extra Detect head) result in measurable performance improvements in commodity detection. Although the approach demonstrates both feasibility and effectiveness in intelligent retail cabinet scenarios, the introduction of multiple enhanced modules (especially the CARAFE module and the extra Detect head) inevitably increases the total number of model parameters and introduces a certain amount of computational overhead. Consequently, the dual objective of significantly improving accuracy and robustness while maintaining or reducing the parameter count has not yet been fully achieved. Future work will focus on more efficient structural optimization strategies, such as lightweight design of the CARAFE module and parameter pruning, aiming to further enhance model performance while maintaining or even reducing computational complexity, making it more suitable for real-time deployment in resource-constrained retail cabinet devices.

**Figure 14:**
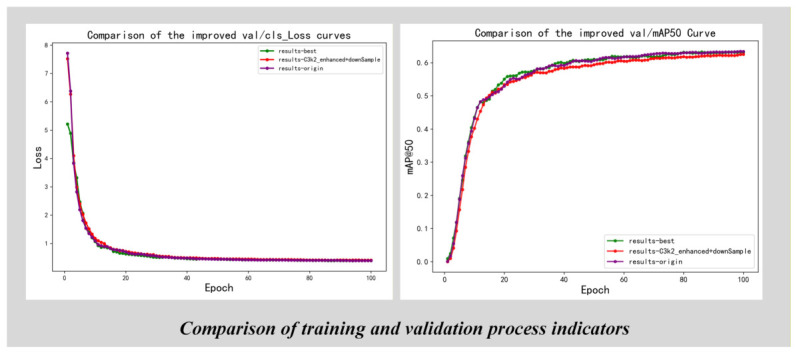
Training and Validation Loss Curves (Epoch = 100).

As illustrated in Figure 14, the improved model (incorporating the CARAFE module and other optimization strategies) outperforms the baseline YOLOv26 model in terms of both convergence speed and final precision. First, in the val/cls_loss curve, the improved model exhibits a faster decline and smaller fluctuations. This is mainly because the C3k2-enhanced module and CARAFE module jointly enhance the feature representation capability, making the model more sensitive to commodity feature differences, thus accelerating the convergence of the classification loss and ensuring a more stable optimization process. Second, in the val/mAP50 curve, the overall performance of the improved model remains consistently above that of the original model and maintains a higher stable value during the middle and later stages of training. This benefit is closely related to the CARAFE module’s ability to enhance feature alignment and the EMA module’s attention guidance, which together improve the model’s detection accuracy and generalization ability, especially for small and occluded commodities.

The main advantages of the proposed model can be summarized as follows:

1. Faster convergence and stronger training stability, benefiting from the enhanced feature extraction of the C3k2-enhanced module and the refined feature alignment of the CARAFE module;
2. Higher final mAP performance, indicating more effective feature extraction and fusion, where the CARAFE module plays a key role in improving the detection accuracy of small objects;
3. Improved robustness in complex scenarios (such as varying illumination and dense multi-commodity), supported by the synergy of data augmentation, EMA module, and CARAFE module.

However, certain limitations also exist. The enhanced architecture, including the introduction of the CARAFE module and extra Detect head, introduces additional structural complexity, which may lead to increased computational overhead and slightly reduced inference speed. Moreover, the performance improvement during the early training stage is not particularly significant, and the model is relatively sensitive to hyperparameter settings (such as the sampling kernel size of the CARAFE module) and training strategies. Therefore, in practical deployment, a careful trade-off between detection accuracy, computational resource consumption, and inference speed is still required.

## 6. Conclusions

The improved object detection model proposed in this study achieves significant enhancements in detection performance, primarily relying on two core innovative designs: a targeted low-light image preprocessing strategy and a synergistic combination of multi-scale downsampling and upsampling. Specifically, aiming at the problem of low detection accuracy under low-light conditions in intelligent retail cabinet scenarios, we innovatively integrate a dedicated low-light preprocessing module into the model pipeline. This preprocessing strategy comprehensively combines adaptive illumination compensation, noise suppression, and detail enhancement operations: it first adjusts the brightness and contrast of low-light images through adaptive histogram equalization to suppress the interference of uneven illumination, then uses a lightweight denoising algorithm to eliminate Gaussian noise generated in low-light image acquisition, and finally enhances the edge and texture details of commodities through guided filtering, ensuring that the subsequent feature extraction module can capture more discriminative information.

In terms of feature processing, we propose an innovative combination of multi-scale downsampling and upsampling, which breaks through the limitations of traditional single-scale sampling or separate up/downsampling operations. Specifically, the C3k2-enhanced module is employed to realize efficient multi-scale downsampling: it strengthens the aggregation of multi-scale feature information during the downsampling process, retains more low-level spatial details and high-level semantic features, and lays a solid foundation for subsequent feature fusion. Meanwhile, the CARAFE content-aware upsampling operator is integrated to complete multi-scale upsampling: unlike traditional bilinear interpolation, CARAFE adaptively assembles local feature patches according to the content of feature maps, effectively solving the problems of feature blurring and detail loss during upsampling. The synergistic combination of these two sampling operations enables the model to flexibly adapt to the scale differences in commodities in retail cabinets (from small snacks to large daily necessities) and significantly improves the detection accuracy of small and occluded targets.

However, the introduction of these two innovative designs, together with the EMA + CONV attention mechanism, inevitably leads to an increase in computational overhead, resulting in a slight decline in inference speed (FPS). In particular, the C3k2-enhanced module, which supports multi-scale downsampling, has more parameters than the original C3k2 structure, and the low-light preprocessing module also introduces a small amount of additional computation, which together exert a certain impact on the real-time performance of the model. To address this limitation, future work will focus on exploring lightweight structural designs for the two core innovative parts: optimizing the low-light preprocessing pipeline to reduce redundant computations while retaining its enhancement effect, and lightweighting the multi-scale sampling combination (e.g., simplifying the channel structure of the C3k2-enhanced module and optimizing the sampling kernel size of the CARAFE module), along with developing more efficient attention mechanisms, aiming to reduce computational complexity while maintaining detection accuracy and improving inference efficiency.

During practical deployment, some targets still exhibit relatively low confidence scores. Further analysis indicates that this issue is mainly attributed to factors such as blurred object boundaries, extreme low-light conditions that exceed the processing range of the preprocessing module, and imbalanced training data distribution. Although our innovative low-light preprocessing strategy has significantly alleviated the impact of uneven illumination, it still has room for improvement in handling extreme low-light scenarios. To mitigate these problems, future efforts may include expanding the diversity of training samples (especially adding more extreme low-light and occluded commodity images), introducing more comprehensive data augmentation strategies (synchronously combining illumination augmentation and occlusion simulation), and performing targeted optimization of the multi-scale sampling combination to enhance the model’s ability to capture details of blurred targets. These improvements are expected to further enhance the robustness and stability of the model under complex environmental conditions.

In addition, to provide a more comprehensive evaluation of the model’s performance, especially the effectiveness of the two core innovative designs, it is recommended to combine qualitative visualization results with quantitative analysis. Specifically, metrics such as category-wise average confidence scores and mean detection accuracy should be considered, along with further evaluation under varying illumination conditions (especially extreme low-light), noise levels, and degrees of image blur. Such fine-grained quantitative analysis not only facilitates a thorough assessment of the model’s adaptability across diverse scenarios but also offers more targeted guidance for the subsequent optimization of the low-light preprocessing module and multi-scale sampling combination.

In summary, although the proposed method, relying on the innovative low-light preprocessing strategy and multi-scale up/downsampling combination, achieves noticeable improvements in detection accuracy (especially in complex low-light and multi-scale commodity scenarios), there remains room for further optimization. Future research will continue to focus on optimizing the low-light preprocessing technique, lightweighting the multi-scale sampling architecture, and developing efficient attention mechanisms, striving to achieve a more favorable balance between detection accuracy, robustness, and real-time performance, making the model more suitable for practical deployment in intelligent retail cabinet edge devices.

## Figures and Tables

**Table 1:** Experiment environment configuration.

Item	Ablation Experiment Configuration	Generalization Experiment Configuration
CPU	Intel(R) Core(TM) i7-14700KF 3.80 GHz	10× Intel(R) Xeon(R) Gold 6248 @ 2.50 GHz
GPU	NVIDIA GeForce RTX 4070Ti (16 GB)	NVIDIA RTX 3080Ti (12 GB)
Deep Learning Framework	CUDA 12.4 + cuDNN v8.9.7	CUDA 12.4 + cuDNN v8.9.7
Framework Version	PyTorch 2.5.1	PyTorch 2.5.1
Operating System	Windows 11 Professional	Ubuntu 22.04 LTS
Image Acquisition Device	HF899 2.7 mm Industrial Camera	–
Development Environment	PyCharm 2025.3	–
Optimization Algorithm	Stochastic Gradient Descent (SGD)	Stochastic Gradient Descent (SGD)
Training Epochs	100	100
Batch Size	16	16
Initial Learning Rate	0.001	0.001
Data Augmentation	Enabled	Enabled
Training Workers	8	10

**Table 2:** Statistics of the self-built dataset.

Dataset	Images	Categories	Split Ratio
Training Set	5167	20	8
Validation Set	638	20	1
Test Set	662	20	1
Total	6467	20	8:1:1

**Table 3:** Performance ccomparison of different baseline models on the public dataset.

Models	GFLOPs	mAP@50	mAP@50–95	Recall	Inference Time (ms)
CenterNet [10]	15.32	0.974	0.628	0.303	4.5
YOLOv8n [26]	8.1	**0.977** *	0.635	0.958	1.7
YOLOv9t [32]	7.6	0.974	0.632	0.954	2.4
YOLOv10n [33]	6.5	0.975	0.633	0.952	2.1
YOLOv11n [31]	6.3	0.976	0.631	0.958	1.7
YOLOv12n [34]	6.3	0.974	0.632	0.955	2.6
YOLOv13n [35]	6.2	**0.975**	**0.636** *	0.955	2.6
RT-DETR-18 [8]	59.3	0.964	0.607	0.954	3.7
YOLOv26 [11]	5.2	0.974	**0.636** *	0.945	**1.5** *
**Ours**	5.4	0.974	0.630	**0.962** *	**1.5** *

* The best performance for each metric is highlighted in bold.

**Table 4:** Performance comparison of different baseline models on the self-constructed dataset.

Models	GFLOPs	mAP@50	mAP@50–95	Recall	Inference Time (ms)
CenterNet [10]	15.32	0.974	0.628	0.953	4.5
YOLOv8n [26]	8.1	**0.977** *	0.635	0.958	1.7
YOLOv9t [32]	7.6	0.974	0.632	0.954	2.4
YOLOv10n [33]	6.5	0.975	0.633	0.952	2.1
YOLOv11n [31]	6.3	0.976	0.631	0.958	1.7
YOLOv12n [34]	6.3	0.974	0.632	0.955	2.6
YOLOv13n [35]	6.2	**0.975** *	**0.636** *	0.955	2.6
RT-DETR-18 [8]	59.3	0.964	0.607	0.954	3.7
YOLOv26 [11]	5.2	0.974	**0.636** *	0.945	1.5
**Ours**	5.4	0.974	0.630	**0.962** *	**1.5** *

* The best performance for each metric is highlighted in bold.

**Table 5:** Attention mechanism comparison experiment.

AttnType	Mean Average Precision@50	Mean Average Precision@50-95	Recall	Giga Floating-Point Operations Per Second	Inference Time (ms)
NO	0.974	**0.636**	0.945	5.8	1.9
CBAM	0.974	0.628	**0.959**	5.8	2.0
CA	0.975	0.629	0.948	5.8	2.0
ECA	0.974	0.627	0.954	5.7	1.8
SENet	0.973	0.633	0.952	5.8	1.8
EMA	**0.976**	0.629	0.953	5.7	1.8

**Table 6:** Multi-module ablation comparison experiment.

Method	C3k2-Enhanced	CARAFE	EMA + CONV	GFLOPs	mAP50	mAP50-95	Recall	Inference Time (ms)
1	×	×	×	5.8	0.974	**0.636**	0.945	1.5
2	✓	✓	×	6.2	**0.976**	0.631	0.952	1.2
3	×	✓	×	6.2	0.971	0.622	0.934	1.3
4	✓	✓	✓	6.3	**0.976**	0.628	**0.954**	1.5

**Note:** Bold values indicate the best performance for each evaluation metric.

## Data Availability

The study utilizes two types of data, and their specific access methods are as follows: 1. Self-constructed data (YZGOODS Dataset): This dataset is independently developed by the author team. The complete dataset and supporting documentation can be accessed via the following link: https://pan.quark.cn/s/3c2adf210705 (accessed on 1 January 2026).

## References

[1] Weinstein R. (2005). RFID: A technical overview and its application to the enterprise. IT Prof..

[2] Xie X., Cheng G., Wang J., Yao X., Han J. (2021). Oriented R-CNN for object detection. Proceedings of the IEEE/CVF International Conference on Computer Vision.

[3] Yang Y. (2024). Vehicle target detection algorithm based on improved Faster R-CNN for remote sensing images. J. Artif. Intell. Pr..

[4] Yang F., Huang L., Tan X., Yuan Y. (2024). FasterNet-SSD: A small object detection method based on SSD model. Signal Image Video Process..

[5] Altarez R.D. (2024). Faster R–CNN, RetinaNet and Single Shot Detector in different ResNet backbones for marine vessel detection using cross polarization C-band SAR imagery. Remote Sens. Appl. Soc. Environ..

[6] Wu Z. (2023). Detect small object based on FCOS and adaptive feature fusion. J. Phys. Conf. Ser..

[7] Zhao Y., Lv W., Xu S., Wei J., Wang G., Dang Q., Liu Y., Chen J. (2024). Detrs beat yolos on real-time object detection. Proceedings of the IEEE/CVF Conference on Computer Vision and Pattern Recognition.

[8] Liu R., Zhang X., Jin S., Wang Q., Zeng L., Liao J. (2024). A small target detection model based on an improved RT-DETR. Proceedings of the 2024 4th International Conference on Industrial Automation, Robotics and Control Engineering (IARCE).

[9] Liao Y.K., Lin G.S., Yeh M.C. (2023). A Transformer-Based Framework for Tiny Object Detection. Proceedings of the 2023 Asia Pacific Signal and Information Processing Association Annual Summit and Conference (APSIPA ASC).

[10] Guo Y., Lu X. (2023). ST-CenterNet: Small target detection algorithm with adaptive data enhancement. Entropy.

[11] Sapkota R., Cheppally R.H., Sharda A., Karkee M. (2025). YOLO26: Key architectural enhancements and performance benchmarking for real-time object detection. arXiv.

[12] Wang S., Xu M., Sun Y., Jiang G., Weng Y., Liu X., Zhao G., Fan H., Li J., Zou C. (2023). Improved single shot detection using DenseNet for tiny target detection. Concurr. Comput. Pract. Exp..

[13] Zhou X., Jiang L., Hu C., Lei S., Zhang T., Mou X. (2022). YOLO-SASE: An improved YOLO algorithm for the small targets detection in complex backgrounds. Sensors.

[14] Jooshin H.K., Nangir M., Seyedarabi H. (2024). Inception-YOLO: Computational cost and accuracy improvement of the YOLOv5 model based on employing modified CSP, SPPF, and inception modules. IET Image Process..

[15] Wang H., Qian H., Feng S. (2024). GAN-STD: Small target detection based on generative adversarial network. J. Real-Time Image Process..

[16] Dong H., Xie K., Xie A., Wen C., He J., Zhang W., Yi D., Yang S. (2023). Detection of occluded small commodities based on feature enhancement under super-resolution. Sensors.

[17] Deng H., Liang P., Liao Z., Yuan J., Wu H. (2025). CAE-YOLOv11n: Research on Commodity Classification for Retail Automated Checkout Systems. Proceedings of the 2025 5th International Conference on Computer Vision, Application and Algorithm (CVAA).

[18] Wang C., Han Y., Yang C., Wu M., Chen Z., Yun L., Jin X. (2025). CF-YOLO for small target detection in drone imagery based on YOLOv11 algorithm. Sci. Rep..

[19] Tang Y., Xu T., Qin H., Li J. (2025). Irstd-yolo: An improved yolo framework for infrared small target detection. IEEE Geosci. Remote. Sens. Lett..

[20] Yue T., Lu X., Cai J., Chen Y., Chu S. (2025). YOLO-MST: Multiscale deep learning method for infrared small target detection based on super-resolution and YOLO. Opt. Laser Technol..

[21] Zhang H., Xiao P., Yao F., Zhang Q., Gong Y. (2025). Fusion of multi-scale attention for aerial images small-target detection model based on PARE-YOLO. Sci. Rep..

[22] Pan Q., Liu Q., Huang W. (2025). NID-DETR: A novel model for accurate target detection in dark environments. Sci. Rep..

[23] Wang W., Yin B., Li L., Li L., Liu H. (2025). A Low Light Image Enhancement Method Based on Dehazing Physical Model. Comput. Model. Eng. Sci. (CMES).

[24] Wadhwa S., Saini P., Kumar R., Kashyap N.S., Siag T. Real time object detection of aerial images using deep learning on Jetson nano. Proceedings of the AIAA SCITECH 2025 Forum.

[25] Tadjine C., Ouafi A., Taleb-Ahmed A., El Hillali Y., Rivenq A. (2025). Object detection based on Logistic Objects in Context (LOCO) dataset: An improved dataset split and performance on NVIDIA Jetson Nano. J. Real-Time Image Process..

[26] Kurniawan A., Ahmad S.F., Wulandari D.P. (2025). Vehicle Speed Estimation System Using Drone With Jetson Nano-based using YOLOv8 Neural Network. Proceedings of the 2025 International Conference on Computer Engineering, Network and Intelligent Multimedia (CENIM).

[27] Iyengar S., Nabavirazavi S., Hariprasad Y., HB P., Mohan C.K. (2025). Real-Time Aquatic Forensics: Harnessing AI for Efficient Underwater Target Recognition. Artificial Intelligence in Practice: Theory and Application for Cyber Security and Forensics.

[28] Wang J., Chen K., Xu R., Liu Z., Loy C.C., Lin D. (2019). Carafe: Content-aware reassembly of features. Proceedings of the IEEE/CVF International Conference on Computer Vision.

[29] Ouyang D., He S., Zhang G., Luo M., Guo H., Zhan J., Huang Z. (2023). Efficient multi-scale attention module with cross-spatial learning. Proceedings of the ICASSP 2023—2023 IEEE International Conference on Acoustics, Speech and Signal Processing (ICASSP).

[30] Wei X.S., Cui Q., Yang L., Wang P., Liu L. (2019). RPC: A large-scale retail product checkout dataset. arXiv.

[31] Khanam R., Hussain M. (2024). Yolov11: An overview of the key architectural enhancements. arXiv.

[32] Zhu J., Ma C., Rong J., Cao Y. (2024). Bird and UAVs recognition detection and tracking based on improved YOLOv9-DeepSORT. IEEE Access.

[33] Li A., Wang C., Ji T., Wang Q., Zhang T. (2024). D3-YOLOv10: Improved YOLOv10-based lightweight tomato detection algorithm under facility scenario. Agriculture.

[34] Khanam R., Hussain M. (2025). A review of YOLOv12: Attention-based enhancements vs. previous versions. arXiv.

[35] Lei M., Li S., Wu Y., Hu H., Zhou Y., Zheng X., Ding G., Du S., Wu Z., Gao Y. (2025). Yolov13: Real-time object detection with hypergraph-enhanced adaptive visual perception. arXiv.

